# The Bright Side and Dark Side of Workplace Social Capital: Opposing Effects of Gender on Overweight among Japanese Employees

**DOI:** 10.1371/journal.pone.0088084

**Published:** 2014-01-31

**Authors:** Tomoko Kobayashi, Etsuji Suzuki, Tuula Oksanen, Ichiro Kawachi, Soshi Takao

**Affiliations:** 1 Department of Epidemiology, Graduate School of Medicine, Dentistry and Pharmaceutical Sciences, Okayama University, Okayama, Japan; 2 Centre of Expertise for Development of Work and Organizations, Finnish Institute of Occupational Health, Helsinki, Finland; 3 Department of Social and Behavioral Sciences, Harvard School of Public Health, Boston, Massachusetts, United States of America; Kyushu University Faculty of Medical Science, Japan

## Abstract

**Background:**

A growing number of studies have sought to examine the health associations of workplace social capital; however, evidence of associations with overweight is sparse. We examined the association between individual perceptions of workplace social capital and overweight among Japanese male and female employees.

**Methodology/Principal Findings:**

We conducted a cross-sectional survey among full-time employees at a company in Osaka prefecture in February 2012. We used an 8-item measure to assess overall and sub-dimensions of workplace social capital, divided into tertiles. Of 1050 employees, 849 responded, and 750 (624 men and 126 women) could be linked to annual health check-up data in the analysis. Binomial logistic regression models were used to calculate odds ratios and 95% confidence intervals for overweight (body mass index: ≥25 kg/m^2^, calculated from measured weight and height) separately for men and women. The prevalence of overweight was 24.5% among men and 14.3% among women. Among men, low levels of bonding and linking social capital in the workplace were associated with a nearly 2-fold risk of overweight compared to high corresponding dimensions of social capital when adjusted for age, sleep hours, physiological distress, and lifestyle. In contrast, among women we found lower overall and linking social capital to be associated with lower odds for overweight even after covariate adjustment. Subsequently, we used multinomial logistic regression analyses to assess the relationships between a 1 standard deviation (SD) decrease in mean social capital and odds of underweight/overweight relative to normal weight. Among men, a 1-SD decrease in overall, bonding, and linking social capital was significantly associated with higher odds of overweight, but not with underweight. Among women, no significant associations were found for either overweight or underweight.

**Conclusions/Significance:**

We found opposite gender relationships between perceived low linking workplace social capital and overweight among Japanese employees.

## Introduction

Obesity and overweight are now a global concern, as an estimated 1.46 billion adults worldwide are overweight or obese [1]. Historically, Japan has had a low prevalence of obesity compared to western populations [2]. However, even in Japan, the rise in obesity and overweight has caused concern, particularly among men and older women [3]. The main cause for obesity is an energy imbalance between intake and expenditure, fuelled by unhealthy behaviors [4] such as the consumption of sugar-sweetened beverages [5], fast food [6], and alcohol [7], physical inactivity [8], short sleep duration [9], as well as individual's genetic disposition [10]. In addition to these factors, there is growing evidence that disadvantaged socioeconomic status [11], [12], as well as adverse working conditions (e.g. shift work [13], long work hours, and psychosocial factors [14]) increases the risk for the overweight.

In 2008, the Japanese Ministry of Health, Labour and Welfare introduced a national strategy to combat obesity and metabolic syndrome. The so-called “Metabo Law” requires companies' health insurers to provide weight loss classes for overweight employees who meet certain criteria at their annual health check-up. If the insurers fail to achieve designated coverage of attending the classes, they are penalized by the government [15]. Although the effectiveness of the new law remains controversial [16], its success may depend on the social cohesion and social capital in the workplace. Although the earliest mention of social capital can be traced back to the beginning of the 20^th^ century [17], it became more formally discussed in the work of Pierre Bourdieu, James S. Coleman, and others [18]. This concept from the fields of sociology, economics, and political science entered the public health literature in the 1990s and has since accumulated a growing evidence base [19]. Social capital is defined as the resources that individuals can access through social networks (e.g. in their neighborhoods, or workplaces) [19]. Such resources can take the form of exchanges of information and expression of solidarity between members. Social networks with more dense ties between members generate more trust as well as reciprocity exchanges, and are hypothesized to be more effective in the maintenance of social norms and their ability to undertake collective action [19].

As an important source of social capital among working populations where people spend a considerable amount of time is the workplace [20], previous studies have examined the relationship between workplace social capital and several health outcomes such as all-cause mortality, hypertension, self-rated health, smoking, depression in Japan [21]–[23], Finland [24]–[29], the Netherlands [30], and China [31]. For example, Suzuki, *et al.* [23] found that lack of individual perceptions of workplace social capital was associated with poor self-rated health in Japan, while no clear associations were found with smoking status [22]. Another Japanese study reported a beneficial effect of workplace social capital on systolic blood pressure [21]. Although most western studies have also consistently suggested beneficial effects of workplace social capital [32], no study has examined its association with overweight. Previous evidence from residential areas in the U.S. suggests that social capital is associated with obesity at state level [33], state or county level [34] as well as neighborhood or regional level [35]. We hypothesized that more socially cohesive workplaces (i.e. workplaces with higher social capital) will be more effective in transmitting norms to keep weight employees at a healthy level. For example, employees in a high social capital workplace may be more likely to encourage their co-workers to stay lean (e.g. by organizing group activities that promote physical activity). On the other hand, by this rationale, socially cohesive workplaces might also exert the opposite effect. For example co-workers might enjoy socializing after hours at drinking parties – or “*nomikai*” – leading to weight gain. “*Nomikai*” parties illustrate workplace social networks, a channel for social support and an occasion to build social capital. Nonetheless, evidence suggests that the risk of overweight might spread in social networks [36].

Besides, research on social capital and health has explored different effects of sub-dimensions of social capital. Previous studies have suggested that social capital of the “bonding” variety (social ties between members who are similar with respect to their social class, gender, age-group, and so forth) can exert a differential effect on health compared to the “bridging” variety (ties between members who are dissimilar in social characteristics) [19]. In the present study, using a multi-dimensional measurement of workplace social capital that has mainly been used in Finnish studies [24], [26], [28], we assessed the associations for overall workplace social capital as well as the three sub-dimensions (bonding, bridging, and linking social capital) [37].

Furthermore, despite the Japanese Equal Employment Opportunity Law enacted in 1986, several traditions have endured, particularly concerning gender roles in the workplace. Career development is completely different for men and women in the typical Japanese workplace. Many women still tend to be employed for non-career-oriented work, and are expected to leave work when they get married or after childbirth [38]. In this patriarchal environment, career-oriented male workers tend to consider themselves as standing apart from women in terms of their identity. Accordingly, we speculated that effects of workplace social capital on overweight might differ by gender.

In the present study, we sought to examine the association between individual perceptions of workplace social capital, as well as its sub-dimensions, and overweight among Japanese employees of a private sector company separately by gender.

## Methods

### Ethics statement

The study received ethical approval from the Ethics Committee on the Research of Epidemiology at Graduate School of Medicine, Dentistry and Pharmaceutical Sciences, Okayama University, and written informed consent was obtained from each participant.

### Participants

We conducted a cross-sectional survey of full-time employees at a company in Osaka in February 2012. Of 1050 employees surveyed, 849 employees aged 18–64 years responded to the questionnaire (response rate 81%). The questionnaire measured individual perceptions of workplace social capital, sociodemographic characteristics, sleep- and health-related behaviors, and psychological distress. Anthropometric data (i.e. weight and height) were collected from the workers' annual health check-ups conducted between June and December 2011. Of 849 respondents, 750 (624 men and 126 women) could be linked to their health check-up data and therefore included in the analysis.

### Measures

We assessed workplace social capital with eight Likert-scaled items (1 = totally disagree, 5 = totally agree). Of the eight items, only the seventh item was measured as follows: 1 = “very little”, 5 = “very much” [24]. We calculated the mean of these eight scores and divided into three categories based on tertile distributions to avoid arbitrary cut off points, because there has been no standard cut off point of social capital to date (combining male and female responses): low (≤3.5), middle (>3.5, ≤4), and high (>4). Furthermore, this measure covered multidimensional aspects including bonding/bridging/linking social capital (see Table S1 for list of items). We also calculated the means for each sub-dimension and divided them into tertiles in the same way as the overall social capital. The categories were: for bonding (≤3.5/>3.5, ≤4/>4), bridging (≤3/>3, ≤4/>4), and linking (<4/4/>4).

Body mass index (BMI) was calculated as weight (kg) divided by the square of height (m^2^). We classified BMI into three categories based on the new criteria of the Japan Society for the Study of Obesity [39]: underweight (<18.5 kg/m^2^), normal body weight (18.5–25 kg/m^2^), and overweight (≥25 kg/m^2^). Although BMI ≥30 kg/m^2^ is defined as obesity by the WHO classification, we combined both overweight and obesity into overweight because of the low prevalence of such obesity in Japan (no more than 2.0% in men and 3.0% in women) [39].

Sociodemographic factors included gender, age (continuous), educational attainment (junior/high school, some college/technical, and college/college graduate) [11], and occupation (clerical, sales, skills, and others) [40]. Sleep- and health-related behaviors included sleep hours (continuous) [4], [9], frequencies of alcohol consumption and physical activity (none/rarely, 1 day/month to 2 days/week, and 3 days/week to almost every day) [4], [7], and smoking status (never/former vs. current) [4]. Psychological distress was assessed by the Japanese version of the Kessler 6 scale (K6), comprising six questions on depression and anxiety [41]. Each question was measured on a 5-point scale and the total score ranged from 0 to 24. We set the cut-off at ≥5 to generate a dichotomous variable in line with previous studies of Japanese population [42].

### Statistical analysis

First, we stratified all analyses by gender, because we found that gender modified the association of workplace social capital and overweight (p = 0.001). Second, we calculated the internal consistency (Cronbach's alpha) and corrected item-total correlations of overall or three sub-dimensions (bonding/bridging/linking) of workplace social capital. Third, we performed a binomial logistic regression analysis to examine the associations between workplace social capital and overweight by combining underweight and normal weight (BMI <25 kg/m^2^). In this analysis, we used the highest tertile of social capital as the referent category and calculated odds ratios (ORs) and 95% confidence intervals (CIs) for overweight. Model 1 included age, and Model 2 additionally adjusted for educational attainment, occupation, sleep hours, frequencies of alcohol consumption and physical activity, smoking status, and the K6 as covariates. Of covariates, categorical variables were included as dummy variables. These analyses were repeated with different sub-dimensions of workplace social capital. We also calculated p values for linear trend by treating the three categories as ordinal variables following a previous study [26]. Fourth, we conducted multinomial logistic regression analysis to estimate ORs and 95% CIs for underweight or overweight relative to normal weight associated with a 1 standard deviation (SD) decrease in mean workplace social capital. By using normal weight as a referent category, we expect that the associations can be examined more clearly than the binominal logistic regression analysis. We considered p values of less than 0.05 (two-tailed) statistically significant. All analyses were performed using STATA 12.1 (StataCorp, College Station, TX, USA).

## Results

The overall Cronbach's alphas were 0.90 for both men and women, and the corrected-item total correlation ranged from 0.62 to 0.75 for men, and from 0.63 to 0.78 for women. The Cronbach's alphas of the three sub-dimensions (bonding/bridging/linking) of workplace social capital were 0.82, 0.88, 0.90 for men and 0.85, 0.89, 0.93 for women, respectively. The corrected-item total correlation of three aspects ranged from 0.62 to 0.86 for men, and from 0.63 to 0.88 for women.

Table 1 shows characteristics of participants by workplace social capital and gender. The overall mean (SD) of workplace social capital was 3.68 (0.62) for men and 3.45 (0.69) for women. The means (SD) of each sub-dimension were: bonding 3.63 (0.68); bridging 3.44 (0.79); linking 3.88 (0.73) for men, bonding 3.39 (0.78); bridging 3.24 (0.78); linking 3.66 (0.86) for women. Of men, 5.1% were underweight and 24.5% were overweight. Of women, 16.7% were underweight and 14.3% were overweight.

**Table 1:** Participants' characteristics and descriptive statistics of workplace social capital, Osaka, Japan (2012).

	Men				Women			
			Workplace social capital				Workplace social capital	
Characteristics	N	%	Mean	SD	N	%	Mean	SD
All	624	100	3.68	0.62	126	100	3.45	0.69
BMI categories								
Underweight	32	5.1	3.76	0.46	21	16.7	3.21	0.58
Normal weight	439	70.4	3.72	0.61	87	69.1	3.47	0.70
Overweight	153	24.5	3.52	0.67	18	14.3	3.66	0.69
Age (years; Means, SD)	36.3	9.57	NA	NA	33.3	7.49	NA	NA
Sleep hours (Means, SD)	5.61	1.06	NA	NA	5.44	1.02	NA	NA
Educational attainment								
Junior high school/high school	108	17.3	3.66	0.52	10	7.9	3.05	0.51
Some college/technical	84	13.5	3.53	0.64	36	28.6	3.30	0.74
College/college graduate	432	69.2	3.71	0.64	80	63.5	3.57	0.65
Occupation								
Clerical	44	7.1	3.55	0.55	33	26.2	3.31	0.65
Sales	43	6.9	3.44	0.87	4	3.2	3.16	0.72
Skills	469	75.2	3.66	0.61	87	69.1	3.50	0.68
Others	68	10.9	3.99	0.44	2	1.6	4.31	0.97
Frequency of alcohol consumption^a^								
None/rarely	157	25.2	3.59	0.70	56	44.4	3.43	0.62
Sometimes	270	43.3	3.74	0.61	60	47.6	3.54	0.74
Often	197	31.6	3.66	0.56	10	7.9	3.09	0.62
Frequency of physical activity^a^								
None/rarely	277	44.4	3.62	0.65	73	57.9	3.35	0.67
Sometimes	316	50.6	3.74	0.6	50	39.7	3.60	0.71
Often	31	5.0	3.49	0.51	3	2.4	3.46	0.19
Smoking status								
Never/former	440	70.5	3.66	0.65	121	96.0	3.47	0.69
Current	184	29.5	3.72	0.55	5	4.0	3.15	0.67
K6 (scores ≥5)								
No	316	50.6	3.79	0.60	59	46.8	3.67	0.57
Yes	308	49.4	3.56	0.62	67	53.2	3.26	0.72

BMI, body mass index; K6, Kessler 6; NA, not applicable; SD, standard deviation.

a Categorized as follows: none/rarely (less than 1 day/month), sometimes (1 day/month to 2 days/week), and often (3 days/week to almost every day).

Table 2 shows associations between workplace social capital and overweight. Among men, although we observed a statistically significant association in the low overall social capital in Model 1 (OR 1.85; 95% CI 1.11–3.08), this association was slightly attenuated in Model 2 (OR 1.65; 95% CI 0.97–2.79). Low bonding and linking social capital were significantly associated with nearly twice the odds of overweight in Model 2 (bonding: OR 1.95; 95% CI 1.06–3.61; linking: OR 1.88; 95% CI 1.13–3.13). Among women, we found that low overall and linking social capital were significantly associated with reduced odds of overweight in Model 2 (overall: OR 0.14; 95% CI 0.03–0.67; linking: OR 0.15; 95% CI 0.03–0.71). Even after adjusting for covariates, all the p values for linear trend were statistically significant in both genders, except for bridging social capital.

**Table 2:** Odds ratios for overweight associated with workplace social capital, Osaka, Japan (2012).

	Men					Women				
		Model 1^a^		Model 2^b^			Model 1^a^		Model 2^b^	
Variables	Overweight/N	OR	(95% CI)	OR	(95% CI)	Overweight/N	OR	(95% CI)	OR	(95% CI)
Workplace social capital										
low: ≤3.5	74/231	1.85	(1.11–3.08)	1.65	(0.97–2.79)	6/63	0.18	(0.04–0.74)	0.14	(0.03–0.67)
middle: >3.5, ≤4	52/257	0.99	(0.59–1.67)	0.92	(0.54–1.58)	7/46	0.35	(0.09–1.39)	0.31	(0.07–1.33)
high: >4	27/136	1.00		1.00		5/17	1.00		1.00	
p for trend		0.005		0.021			0.021		0.016	
Bonding social capital										
low: ≤3.5	74/231	2.17	(1.19–3.95)	1.95	(1.06–3.61)	5/60	0.25	(0.04–1.65)	0.18	(0.02–1.45)
middle: >3.5, ≤4	62/298	1.16	(0.64–2.11)	1.13	(0.61–2.06)	11/56	0.81	(0.14–4.65)	0.59	(0.09–3.95)
high: >4	17/95	1.00		1.00		2/10	1.00		1.00	
p for trend		0.001		0.007			0.043		0.039	
Bridging social capital										
low: ≤3	69/258	2.39	(0.97–5.89)	2.13	(0.85–5.31)	9/67	NA		NA	
middle: >3, ≤4	78/320	2.04	(0.83–5.02)	1.95	(0.79–4.82)	9/55	1.39	(0.50–3.85)	1.44	(0.46–4.45)
high: >4	6/46	1.00		1.00		0/4	1.00		1.00	
p for trend		0.082		0.185			0.818		0.819	
Linking social capital										
low: <4	67/204	1.94	(1.18–3.20)	1.88	(1.13–3.13)	5/59	0.25	(0.06–0.94)	0.15	(0.03–0.71)
middle: 4	56/261	1.08	(0.66–1.79)	1.07	(0.64–1.79)	7/40	0.62	(0.17–2.20)	0.53	(0.13–2.17)
high: >4	30/159	1.00		1.00		6/27	1.00		1.00	
p for trend		0.004		0.008			0.035		0.014	

CI, confidence interval; NA, not applicable; OR, odds ratio.

a Adjusted for age.

b Adjusted for age, sleep hours, educational attainment, occupation, frequencies of alcohol consumption and physical activity, smoking status, and K6 scores.

Table 3 shows the results of multinomial logistic regression analysis to estimate relationships of workplace social capital for being underweight or overweight relative to normal weight. Among men, a 1-SD decrease in mean of workplace social capital was significantly associated with higher odds of overweight, except for bridging social capital, and no significant associations were found for underweight. Among women, neither the overall nor any sub-dimensions of workplace social capital were significantly associated with either overweight or underweight.

**Table 3:** Odds ratios for underweight/overweight per a 1-SD decrease in the mean of workplace social capital relative to normal weight, Osaka, Japan (2012).

	Men				Women			
	Model 1^a^		Model 2^b^		Model 1^a^		Model 2^b^	
	OR	(95% CI)	OR	(95% CI)	OR	(95% CI)	OR	(95% CI)
Workplace social capital								
Underweight	0.92	(0.62–1.36)	0.90	(0.60–1.34)	1.38	(0.86–2.21)	1.40	(0.82–2.38)
Normal weight	1.00		1.00		1.00		1.00	
Overweight	1.39	(1.16–1.67)	1.35	(1.11–1.63)	0.73	(0.42–1.27)	0.59	(0.30–1.13)
Bonding social capital								
Underweight	1.00	(0.69–1.47)	0.98	(0.66–1.46)	1.31	(0.83–2.07)	1.26	(0.77–2.07)
Normal weight	1.00		1.00		1.00		1.00	
Overweight	1.38	(1.16–1.65)	1.37	(1.13–1.65)	0.78	(0.45–1.36)	0.68	(0.36–1.29)
Bridging social capital								
Underweight	1.08	(0.75–1.55)	1.04	(0.72–1.52)	1.25	(0.79–1.97)	1.30	(0.77–2.18)
Normal weight	1.00		1.00		1.00		1.00	
Overweight	1.18	(0.98–1.41)	1.15	(0.96–1.39)	0.95	(0.56–1.60)	0.86	(0.46–1.62)
Linking social capital								
Underweight	0.76	(0.50–1.15)	0.76	(0.49–1.16)	1.31	(0.84–2.05)	1.35	(0.82–2.22)
Normal weight	1.00		1.00		1.00		1.00	
Overweight	1.38	(1.15–1.65)	1.32	(1.10–1.59)	0.63	(0.35–1.15)	0.50	(0.24–1.02)

CI, confidence interval; OR, odds ratio; SD, standard deviation.

a Adjusted for age.

b Adjusted for age, sleep hours, educational attainment, occupation, frequencies of alcohol consumption and physical activity, smoking status, and K6 scores.

## Discussion

Our findings suggest that men reporting low bonding or linking workplace social capital had increased odds of overweight, whereas women reporting low overall or linking social capital had decreased odds of overweight. Notably, we found a gender difference in the direction of associations between low linking social capital and overweight. Among men, a 1-SD decrease in overall, bonding, and linking social capital was significantly associated with higher odds of overweight, but not with underweight. Among women, no significant associations were found for either overweight or underweight. Our findings indicate that social capital can have bright and dark sides in Japanese workplaces in terms of employee health.

While to date no previous studies in an occupational setting have reported associations between overall workplace social capital and overweight/obesity, our results among women are not consistent with the previous findings that higher social capital in a community setting is inversely associated with obesity in the United States and England [33]–[35]. Furthermore, in a Finnish cross-sectional study, women reporting low overall workplace social capital had increased odds of poor self-rated health [24]. Therefore, in contrast to these previous findings, our study implies the presence of a dark side of workplace social capital among women. Among Japanese men, strong social solidarity in the workplace is sometimes expressed in the form of informal ‘social drinking’ after work, known as “*nomikai*”. As reported in a study conducted in 1993–94 [43], middle-aged Japanese men spend several nights a week with male colleagues to relieve stress and to build solidarity. As a result, men reporting strong social support at work appeared to engage in heavier drinking and also reported a higher fat intake pattern, potentially leading to overweight. In contrast to men, the pooled analyses of six Japanese cohort studies have shown that the prevalence of alcohol drinkers among women was quite low compared to male workers [44], and social drinking is also less likely to be common among women in the workplace. In this study, only 7.9% among women reported that they consume alcohol often. Therefore, the “*nomikai*” effect is not likely to be a plausible explanation for the dark side of workplace social capital among women.

Previous studies tend to suggest that social capital of the “bonding” variety can exert a differential effect on health compared to the “bridging” variety [19]. In a cross-sectional study among residents of a disadvantaged, predominantly minority community in the United States, the researchers suggested that high bonding social capital was associated with higher levels of mental distress [45]. On the other hand, a recent cross-sectional study in two Dutch companies suggested that bonding social capital was positively associated with better self-rated health. In addition, Kim *et al* [46] reported protective effects of community bonding social capital on self-rated health within communities in the United States. With regard to bonding social capital, our findings among men are consistent with these previous studies that suggested beneficial effects of bonding social capital. In contrast to bonding social capital, we found no significant associations between bridging social capital and overweight either among men or women. Although a population-based study in Japan suggests that bridging social capital was significantly associated with better self-rated health [47], especially among women, further studies are needed to examine possible protective effects of bridging social capital on overweight in workplaces. Linking social capital might yield health benefits by connecting people across “vertical” different authority gradients [37]. Our findings among men appear to be in line with some evidence showing that linking workplace social capital was inversely associated with emotional exhaustion [30]. However, among women, we found that the pattern was opposite to men, i.e., low linking social capital was associated with reduced risk of overweight. The reason for this gender difference is not clear; the empirical evidence for linking social capital in the workplace remains too sparse to draw generalizations. Further research is needed to investigate the potential gender difference the relation between linking social capital and workers' health.

With regard to the opposing effects of gender on the association between workplace social capital and overweight, a possible explanation may be that women are affected more than men by factors outside work [48]. For example, a study suggested that women are influenced more than men from higher levels of neighborhood social capital [49]. If women with low linking workplace social capital were more likely to compensate higher levels of neighborhood social capital, and neighborhood social capital had protective effects on overweight like previous studies [33], [34], in that case, our inability to measure neighborhood social capital could have influenced our findings among women due to exposure misclassification. Further studies are warranted to examine these possible work-community (outside work) interactions.

When we analyzed the data by using multinominal logistic regression models, the results were consistent with the results of binominal logistic regression models – i.e. a 1-SD decrease in bonding and linking social capital were associated with increased odds of overweight among men. A possible novel aspect of multinominal regression model is each 1 SD decrease in workplace social capital was associated with between 26–40% increased odds of underweight in women. Although these estimates were not statistically significant, this tendency warrants future investigation and corroboration.

## Strengths and Limitations

This is the first study to examine the association of workplace social capital and its sub-dimensions and overweight. The response rate was high, BMI was based on health check-up data rather than self-report, and we assessed sub-dimensions of workplace social capital in addition to the overall score. However, several limitations should be noted. First, although we examined the relationship between individual perceptions of workplace social capital and overweight, an ideal exploration of the effect of social capital on health requires measurement of the construct at both the individual and the collective level (e.g. work unit or company level), implemented within a multi-level analytical framework. The fact that we relied exclusively on individual perceptions means that some of our findings could have been “contaminated” by individual differences in negative affectivity, attitude towards work, social desirability, and so on. For example, some evidence has shown that obese persons are more likely to report job-related discrimination and lower levels of self-acceptance than normal weight persons [50]. That is, overweight could result in stigma and ostracism from the group, and hence result in lower perceptions of workplace social capital. Second, the cross-sectional study design cannot establish causation. We cannot rule out the possibility that the 3–8 months time lag between collection of BMI and social capital data may have influenced the results of the present study, i.e. our findings reflect a degree of reverse causation (workers who gain weight perceive their workplaces as less cohesive). A stronger design would be to examine changes in body weight in relation to changes in social capital perceptions within a fixed effects framework. Third, the prevalence of overweight among men was nearly twice than among women. Thus, the observed gender differences in this study may reflect this type of selection process. Fourth, our study mainly included skilled workers from a private company, limiting the generalizability of our findings to the total labor force. Fifth, the possibility of residual confounding cannot be ruled out since the information about some potential prior common causes of workplace social capital and overweight was not available. For example, attendance at after-work drinking sessions (*nomikai*) varies by the worker's marital status and presence of children at home [51], [52]; hence these variables affect both the level of workplace social capital (via informal socializing with co-workers) as well as risk of overweight. Finally, because of small numbers and relatively narrow distributions of especially bridging social capital among women, we could not explore these associations in greater depth. In addition, as our study sample was of uniform race/ethnicity and had relatively small variations in social class indicators (all participants worked in the same company), it is possible that we failed to capture bridging social capital differently from bonding/linking social capital.

## Conclusions

The present study suggests that gender differences exist in the associations between low linking workplace social capital and overweight among Japanese employees. These results lend support to the notion that social capital has both a bright and a dark side. Further studies should examine the possible link between workplace social capital and overweight to elucidate the reason for the differences based on gender.

## Supporting Information

Table S1 **8 items used to measure workplace social capital.**
(PDF)
