# Supplementary material for: The Bright Side and Dark Side of Workplace Social Capital: Opposing Effects of Gender on Overweight among Japanese Employees
Source: PLoS One. 2014 Jan 31;9(1):e88084. doi: 10.1371/journal.pone.0088084 (PMC3909277; doi:10.1371/journal.pone.0088084)
Supplement: Table S1 — 8 items used to measure workplace social capital. (PDF) [file pone.0088084.s001.pdf]

Table S1. 8 items used to measure workplace social capital

Items	Bonding/bridging/linking
1. Our supervisor treats us with kindness and consideration.	Linking
2. Our supervisor shows concern for our rights as an employee.	Linking
3. We have a 'we are together' attitude.	Bonding
4. People keep each other informed about work-related issues in the work unit.	Bonding
5. People feel understood and accepted by each other.	Bonding
6. Do members of the work unit build on each other's ideas in order to achieve the best possible outcome?	Bridging
7. People in the work unit cooperate in order to help develop and apply new ideas.	Bridging
8. We can trust our supervisor.	Linking

The responses were given in a 5-point Likert-scale (1=”totally disagree”, 5=”totally agree”), except for the seventh item in which the categories were: 1=”very little”, 5=”very much”.
